# Study on the Effects of Combined Exposure to a High-Fat, High-Sugar Diet and a High-Temperature, High-Humidity Environment on Adaptive Immunity in CHB Mice Based on Multi-Omics Analysis

**DOI:** 10.3390/biology15201811

**Published:** 2026-10-10

**Authors:** Qiuping Chen, Haiyang Hu, Yichen Peng, Haihui Liu, Yuming Jiang, Quansheng Feng, Baixue Li

**Affiliations:** 1 School of Basic Medical Sciences, Chengdu University of Traditional Chinese Medicine, Chengdu 611137, China; chenqiuping@stu.cdutcm.edu.cn (Q.C.);; 2 Department of Gastroenterology, The First Medical Center, Chinese PLA General Hospital, Beijing 100853, China; 3 Key Laboratory of Digital Protection for Unearthed Medical Literature and Cultural Relics, Chengdu University of Traditional Chinese Medicine, Chengdu 611137, China; jiangym@scu.edu.cn; 4 School of Computer Science (School of Software, School of Intelligent Science and Technology), Sichuan University, Chengdu 610207, China

**Keywords:** chronic hepatitis B, high-fat, high-sugar, high-temperature, high-humidity, miR-340-5p, Psat1, CD4^+^ memory T cells

## Abstract

(1) Background: Chronic hepatitis B (CHB) is a widely prevalent chronic liver disease, with a particularly high incidence in China. Recent studies indicate that external influences like a high-fat, high-sugar diet and high temperature and humidity may alter the hepatic immune microenvironment in CHB and hasten liver injury progression in patients. Nonetheless, the cumulative effect of these combined factors on liver damage and the immune remodeling process, along with its regulatory mechanisms, remains uncertain. (2) Objective: This research seeks to clarify the impact of simultaneous exposure to a high-fat, high-sugar diet and a high-temperature, high-humidity environment on CHB liver injury and its primary immunometabolic pathways. (3) Methods: This research employed recombinant adenovirus AdrcccDNA to establish a CHB mouse model (M group), and subsequently developed a combined exposure model through intervention with a high-fat, high-sugar diet and a high-temperature, high-humidity environment (NWS group). Subsequently, multi-omics analysis, WGCNA, immune infiltration analysis, and in vivo and in vitro experiments were employed to evaluate the effects of a high-fat and high-sugar diet combined with high-temperature and high-humidity exposure on the progression of CHB. (4) Results: HE staining, virology, and other results showed that combined exposure worsened liver injury and increased viral replication in CHB mice. WGCNA and immune infiltration analysis revealed CD4^+^ T cells as the key differential immune cells and identified 36 associated core mRNAs. Combined analysis revealed that miR-340-5p was significantly negatively correlated with Psat1 expression and highly associated with the proliferation and function of memory CD4^+^ T cells. In vivo validation indicated that in the NWS group, Psat1 levels were reduced, miR-340-5p levels were elevated, and the percentage of memory CD4^+^ T cells was diminished. After in vitro transfection with miR-340-5p mimics, Psat1 levels were downregulated in AML12 and primary CD4^+^ T cells, indicating that miR-340-5p negatively influences Psat1. (5) Conclusions: This study demonstrates that combined exposure to a high-fat, high-sugar diet and a high-temperature, high-humidity environment may worsen CHB liver injury through miR-340-5p/Psat1-mediated immunometabolic regulation.

## Simple Summary

Chronic hepatitis B is a common liver disease, especially in China. Beyond the virus itself, lifestyle and environmental factors—such as a high-fat, high-sugar diet and hot, humid weather—may also affect how the disease develops. However, it is still unclear how these factors work together to worsen liver damage. This study aimed to investigate how combined exposure to a high-fat, high-sugar diet and a high-temperature, high-humidity environment affects liver injury in chronic hepatitis B and to identify the underlying mechanism. We built a mouse model of chronic hepatitis B and exposed the mice to these conditions. We found that the combined exposure made liver injury worse and increased virus replication. Using large-scale molecular analyses and experiments, we identified a key regulatory pair: a small RNA molecule called miR-340-5p and its target gene Psat1. The combined exposure raised miR-340-5p levels, which lowered Psat1 levels and reduced an important type of immune cell. These findings suggest that a high-fat, high-sugar diet plus a hot, humid environment may worsen chronic hepatitis B through this pathway. This work may help identify people at higher risk and inform prevention strategies.

## 1. Introduction

With global warming intensifying and Earth’s mean temperature climbing notably, the amount of water vapor in the atmosphere is rising at the same rate. Research indicates that a 1 °C rise in temperature corresponds to an approximate 7% increase in atmospheric water vapor content [1]. The Intergovernmental Panel on Climate Change (IPCC) has indicated that since the 1950s, global warming has led to an ongoing increase in the frequency, duration, and intensity of heatwaves [2,3,4,5]. Heatwaves are typically prolonged periods of abnormally high temperatures, often accompanied by high humidity, exerting widespread impacts on human health and social systems. Recently, multiple large-scale, high-intensity extreme heatwave events have taken place in the Northern Hemisphere [6,7]. Future heatwaves will still occur more often and with greater force, even if the rise in global temperature is kept under 1.5 °C. Numerous epidemiological studies indicate that heatwave exposure significantly raises overall morbidity and mortality rates and is linked to fluid and electrolyte imbalances, impaired renal function, and an increased risk of liver cancer mortality [8,9,10,11]. A study involving 1943 counties in the United States indicated that during heatwaves, fluid and electrolyte disorders increased by 18%, kidney failure increased by 14%, and the incidence of heatstroke increased by 154% [12]. According to studies conducted in China’s Shandong Province, heatwaves are linked to a 3.5-fold rise in the likelihood of dying from liver cancer [13]. It is worth noting that some studies suggest that hot and humid environments may not only increase the risk of hepatitis B virus (HBV) infection but also promote the progression of chronic hepatitis [14,15].

Besides severe changes in climate, there is a significant transformation in global eating habits, characterized by an increasing consumption of calorie-dense items like those high in fat and sugar [16]. A diet high in fat and sugar is widely acknowledged as a significant risk factor for metabolic dysfunction and progression of chronic liver disease. Research indicates that a diet high in fat and sugar can lead to lipid metabolism disorders, chronic inflammation, and oxidative stress, which may contribute to liver fibrosis and hepatocellular carcinoma [17,18,19,20]. Therefore, high-temperature and high-humidity environments, combined with diets rich in fat and sugar, are important external exposure factors commonly faced by people around the world today. Nonetheless, there remains insufficient research on whether the combined exposure to these factors facilitates CHB progression by altering the host immune microenvironment, along with its underlying molecular mechanisms.

Characterized as a double-stranded circular DNA virus with a preference for liver cells, the HBV creates a substantial global public health burden through its infections [21]. At present, roughly 2 billion individuals across the globe have contracted HBV, with almost 300 million suffering from chronic infections and around 820,000 annual fatalities caused by HBV-associated cirrhosis and liver cancer [22,23]. It has been established by various studies that persistent HBV infection stimulates hepatic stellate cells and fosters abnormal extracellular matrix buildup, which can progress to liver fibrosis, cirrhosis, and the occurrence of HCC [24,25,26]. Despite significant reductions in disease burden from HBV vaccination and antiviral therapy, achieving a functional cure remains challenging, with only a small number of patients sustaining long-term HBV DNA negativity [27,28,29,30,31,32]. The primary reason is that covalently closed circular DNA (cccDNA) in infected hepatocytes remains stable for an extended period, continuously acting as the template for viral replication and transcription. Currently, growing evidence suggests that liver damage related to HBV is primarily caused by prolonged immune dysregulation and chronic inflammation rather than direct viral action [33].

It is important to highlight that HBV specifically targets human hepatocytes, and standard mice cannot be naturally infected with HBV. For a long time, the absence of animal models that can maintain both persistent viral replication and a functional host immune response has hindered in-depth studies on the mechanisms of chronic HBV infection and immune regulation. Recently, Deng Qiang and colleagues utilized an adenovirus vector to introduce rcccDNA into the liver of mice, creating a model of chronic hepatitis with ongoing infection by suppressing host immunity and promoting epigenetic silencing of cccDNA [33]. This model replicates the biological characteristics of chronic HBV infection and mimics the pathophysiological process of chronic hepatitis in immunocompetent mice, offering a stable and dependable in vivo platform for this study. Consequently, this research created a composite exposure CHB mouse model based on mice with persistent HBV rcccDNA infection, combined with a high-fat and high-sugar diet and a high-temperature and high-humidity environment. We further integrated miRNAome, mRNA transcriptome, and proteomics data, combined with WGCNA, immune infiltration analysis, and experimental validation, to identify key immune cell subpopulations and regulatory molecules. We aimed to clarify the molecular mechanisms through which a high-fat and high-sugar diet, along with a damp-heat environment, contributes to liver injury during chronic HBV infection.

## 2. Materials and Methods

### 2.1. Construction of the Animal Model

**Figure 1:**
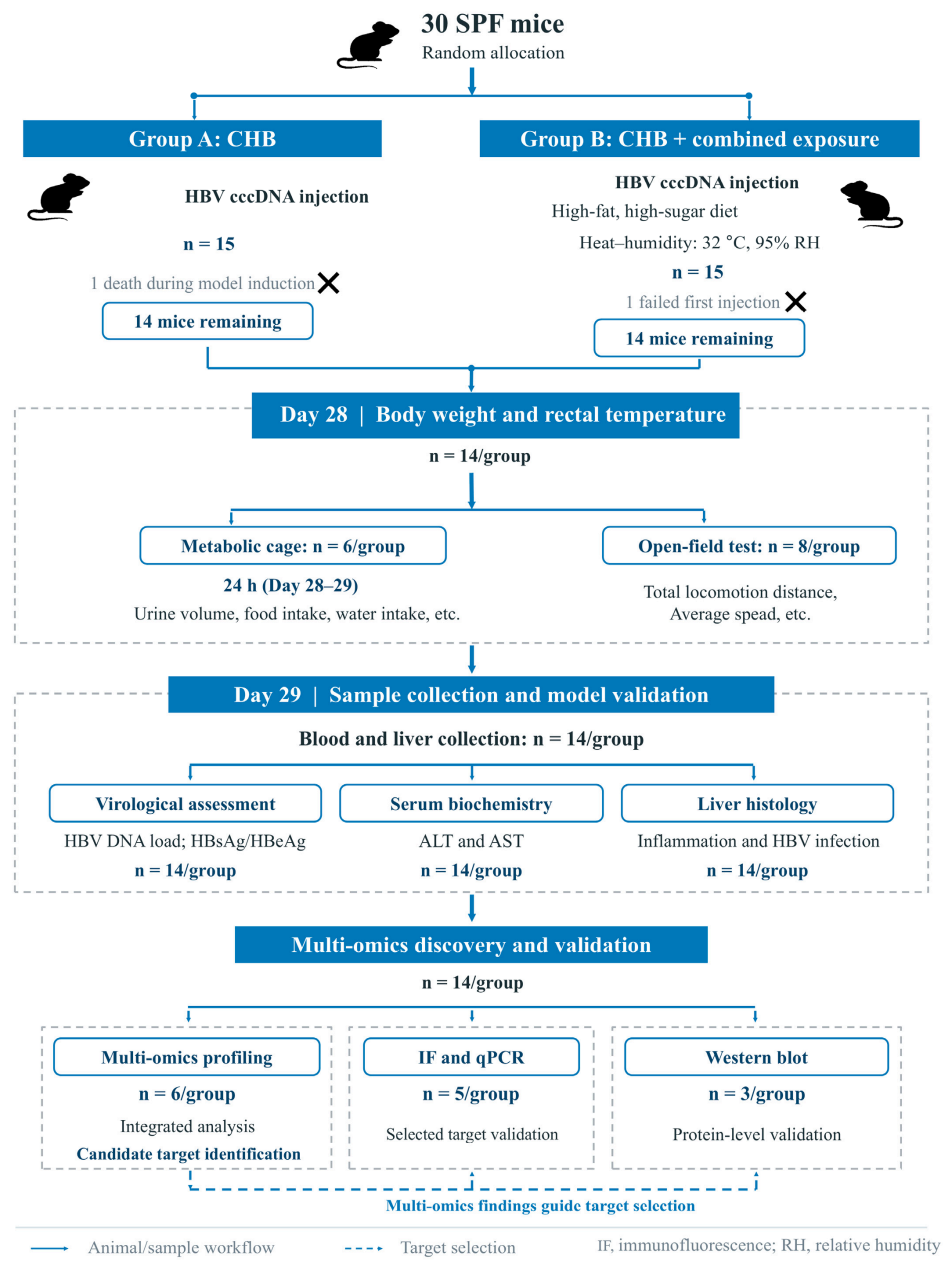
Schematic overview of the experimental design and workflow.

Thirty SPF-grade female *Alb-Cre* mice (6–8 weeks old, weighing 16.0–20.0 g) were purchased from Guangzhou Bojin Biotechnology Co., Ltd. (Guangzhou, China) [Laboratory Animal Use License No.: SYXK (Yue) 2023-0343] and housed at the Animal Experiment Center of Chengdu University of Traditional Chinese Medicine. Animals were maintained at 25 °C with 50–60% relative humidity, a 12 h light/12 h dark cycle, and had free access to food and water. All mice were acclimatized for 7 days before the experiment was conducted. The animal experimental protocol was approved by the Experimental Animal Ethics Committee of Chengdu University of Traditional Chinese Medicine (Approval No.: 2022-31), and all experimental procedures were performed in accordance with the relevant institutional ethical guidelines for animal experiments. After the adaptive feeding period, the mice included in the experiment were randomly divided into a chronic HBV infection model group (group M) and a chronic HBV infection combined with high-fat and high-sugar diet and high-temperature and high-humidity environment intervention group (group NWS). Both groups of mice received an injection of Ad/rcccDNA (1.5 × 10^9^ PFU per mouse, injection volume 200 μL) via the tail vein to create a persistent HBV infection model. On day 7 after injection, peripheral blood was collected via the retro-orbital venous plexus to measure HBsAg levels and determine whether the model was successfully established. Mice in group M continued to be housed under standard environmental conditions and were given regular maintenance feed and drinking water. Mice in the NWS group received a high-fat and high-sugar diet combined with high-temperature and high-humidity environment exposure intervention. Specifically, mice were given a 25% high-fat diet and allowed free access to a 160 g/L glucose solution, while being housed in a climate-controlled chamber at 32 °C and 95% relative humidity. Both groups of animals had free access to food and water, and the intervention lasted for 4 weeks. After the intervention, terminal experiments were performed on the mice and they were sacrificed, with blood and liver tissue collected. After processing, the blood was utilized to extract serum for further biochemical and related indicator testing; the liver tissue was divided, preserved, or frozen based on the objectives of future experiments in histopathology, molecular biology, and multi-omics analysis. Table 1 displays the composition, content, and energy supply ratio of the high-fat diet compared to the standard maintenance diet, while Figure 1 illustrates the overall flow of the animal experiment.

### 2.2. General Observations and Open-Field Test

All mice had their rectal temperatures measured using a rectal thermometer on days 1, 7, 14, 21, and 28. After the 28th day of rectal temperature measurement, 6 animals were selected from each group and individually placed in metabolic cages, and the 24-h food intake, water intake, and urine volume were recorded. Food intake and water intake were calculated as the initial weight of pre-weighed standard diet and water bottles minus the remaining weight after 24 h, respectively. Additionally, 8 mice from each group were subjected to the open-field test in a quiet environment. Each mouse was placed in the center of the open field and allowed to move freely for 5 min. The VisuTrack rodent behavioral video analysis system was used to record and analyze parameters including total distance traveled (mm), average speed (mm/s), rearing frequency, and immobility time (s).

### 2.3. Hematoxylin and Eosin (H&E) Staining of Hepatic Tissues

Fresh mouse liver tissue was fixed in 4% paraformaldehyde for 24 h, followed by sequential gradient ethanol dehydration, clearing, paraffin embedding, and sectioning. Tissue sections were first deparaffinized in xylene, rehydrated through a graded ethanol series, and then subjected to hematoxylin and eosin (H&E) staining. After staining, the sections were dehydrated through a graded ethanol series, cleared in xylene, and mounted with neutral balsam. H&E-stained sections were subjected to histopathological observation under 100× and 400× microscopes, and representative images were captured. Meanwhile, whole-slide TIFF digital images were acquired using a high-resolution pathology scanner. Histological evaluation was independently assessed by two blinded pathologists. Histological scoring was performed using the modified Knodell inflammatory activity semiquantitative scoring system, assessing portal inflammation (P), lobular inflammation (L), piecemeal necrosis (PN), and bridging necrosis (BN), respectively. The total score was calculated according to the weighted formula P + L + 2 × (PN + BN). Scoring was performed independently by two pathologists, and any discrepancies were resolved through discussion. ImageJ software (version 1.54p, National Institutes of Health, Bethesda, MD, USA) was used to quantitatively evaluate the infiltration of inflammatory cells.

### 2.4. Measurement of Serum HBsAg, HBeAg, IL-6, TNF-α, ALT, AST, and Corticosterone

All ELISA tests were performed strictly in accordance with the kit manufacturer’s instructions. IL-6 (Reed Biotech, RE3186M, Wuhan, China) and TNF-α (Reed Biotech, RE1060M) were detected using liver tissue homogenate samples. Other indicators, including HBsAg (Shanghai Source Orange, YJ34199, Shanghai, China), HBeAg (Shanghai Source Orange, YJ688541), ALT (Shanghai Source Orange, YJ35372), AST (Shanghai Source Orange, YJ35235), and Corticosterone (Elabscience, E-OSEL-M0001, Wuhan, China), were measured using peripheral blood serum samples. Absorbance was measured at 450 nm using a SpectraMax Plus spectrophotometer (Molecular Devices, San Jose, CA, USA).

### 2.5. Hepatic HBV DNA Loads Were Determined by Quantitative Real-Time PCR

Liver tissue samples (20 mg) were collected, frozen in liquid nitrogen, and ground into a powder before being placed in 1.5-mL centrifuge tubes. Lysis, column purification, and elution were performed according to the instructions provided with the Tiangen Genomic DNA Extraction Kit (DP304, Beijing, China), with the final elution performed using 50 µL of nuclease-free water to obtain genomic DNA. A 1 µL aliquot of the DNA template was subsequently added to a 20 µL qPCR reaction system for amplification, while the remaining DNA was stored at −80 °C. The primer sequences used for qPCR are listed in Table 2.

### 2.6. RNA Extraction, Sequencing, and Bioinformatics Analysis of Liver Tissues

Total RNA was extracted from approximately 50 mg of liver tissue using the TRIzol method. RNA concentration and purity were measured using a NanoDrop ND-1000 spectrophotometer (NanoDrop Technologies, Wilmington, DE, USA), with an A260/A280 ratio of 1.8–2.0 and an A260/A230 ratio > 2.0 required. RNA integrity was assessed using the Agilent 2100 Bioanalyzer (Agilent Technologies, Inc., Santa Clara, CA, USA), with a requirement of RIN ≥ 7.0. The mRNA and miRNA libraries were constructed by Lianchuan Biotechnology Co., Ltd. (Lianchuan Biotechnology Co., Ltd., Hangzhou, China). The mRNA library was sequenced on the Illumina NovaSeq 6000 (Illumina, Inc., San Diego, CA, USA) platform using a 150 bp paired-end (PE150) protocol. The miRNA library was sequenced on the Illumina HiSeq 2500 platform using a 50 bp single-end protocol [34].

The raw mRNA sequencing data were processed with Cutadapt (version 1.9) to remove adapter sequences and low-quality reads, and FastQC (version 0.11.9) was used for quality control to obtain clean reads. Clean reads were aligned to the mouse reference genome using HISAT2 (version 2.2.1). Transcript assembly was performed using StringTie (version 2.1.6), and gffcompare (version 0.9.8) was used to compare with the reference genome annotation, classify known transcripts, and identify novel transcripts. Based on reference genome annotation, the FPKM and read count expression profiles of protein-coding gene mRNAs were calculated. Differential expression analysis used read counts as input and employed DESeq2 or edgeR based on the negative binomial distribution model. When sufficient biological replicates were available, DESeq2 was preferred; for other experimental designs, edgeR was used. The screening threshold for differential mRNAs was |log2FC| ≥ 1 and *p* < 0.05 (or FDR < 0.05). Differential mRNAs were visualized using bar charts, volcano plots, and clustering heatmaps (Z-score standardization).

The raw sequencing data were quality-controlled to obtain clean data, and high-quality reads were further screened. High-quality reads were annotated using databases such as Rfam and Repbase to remove non-miRNA sequences including rRNA, tRNA, snoRNA, and repetitive sequences. The remaining reads were compared against the miRBase database to identify known miRNAs; the unaligned reads were subjected to hairpin structure prediction to identify novel miRNAs. Expression quantification and differential analysis were performed on the identified miRNAs. For differential analysis, methods such as Fisher’s exact test, chi-square test, *t*-test, or ANOVA were selected according to the experimental design. When biological replicates were present, *p* < 0.05 was used as the threshold for significant difference; when biological replicates were absent, |log2FC| ≥ 1 and *p* < 0.05 were used as the threshold for significant difference. Differential miRNAs were visualized using bar charts, volcano plots, and clustering heatmaps. For differentially expressed miRNAs, TargetScan [35] and miRanda [36] were used for target gene prediction. After screening according to their respective scoring criteria, the intersection was taken as the final target genes.

### 2.7. Proteomic Sequencing and Bioinformatics Analysis

Liver tissue was homogenized in lysis buffer (8 M urea, 50 mM Tris-HCl, and Roche protease inhibitor cocktail) and centrifuged at 4 °C. The supernatant was taken and reduced with DTT (37 °C, 1 h), then alkylated with IAA (room temperature, 30 min, protected from light). Protein quantification and quality control were performed using the Bradford method and SDS-PAGE, respectively. An aliquot of protein (150 µg) was taken and digested with trypsin (enzyme/substrate ratio 1:50) at 37 °C for 14–16 h. Each sample peptide was desalted, dried, and reconstituted, after which equal amounts were taken for high-pH reversed-phase chromatography fractionation, which was performed on a Thermo UltiMate 3000 system (Thermo Fisher Scientific, Waltham, MA, USA) using a ZORBAX C18 column (Agilent Technologies, Inc., Santa Clara, CA, USA). After lyophilization and reconstitution, each fraction was analyzed on an EASY-nLC 1200 system coupled to an Orbitrap Exploris 480 mass spectrometer in DIA mode (Thermo Fisher Scientific, Waltham, MA, USA). The raw data were searched against a protein database using DIA-NN (v1.8) in Direct DIA mode, with trypsin/P as the enzyme cleavage specificity. Carbamidomethylation of C was set as a fixed modification, oxidation of M and N-terminal acetylation were set as variable modifications. The FDR at both the peptide and protein levels was <1%. Protein intensities were normalized by the median, and differentially expressed proteins (DEPs) were defined as |log2FC| ≥ 0.26 and *p* < 0.05. Downstream bioinformatics analysis was performed using the online platform provided by Hangzhou Lianchuan Biotechnology Co., Ltd. (Hangzhou, China).

### 2.8. Weighted Gene Co-Expression Network Analysis (WGCNA) and CIBERSORT Immune Cell Deconvolution

Outlier samples were initially excluded through hierarchical clustering analysis based on the gene expression data from all samples. A soft-thresholding parameter β was utilized to weight the Pearson correlation coefficients, with the optimal value determined based on the scale-free network fitting criterion. A topological overlap matrix (TOM) was generated, and gene co-expression modules were identified using hierarchical clustering and the dynamic hybrid cut algorithm, with a minimum module size of 30 genes and a dissimilarity threshold of 0.25 for merging modules. The correlation between module eigengenes and sample traits was calculated to screen for key modules associated with phenotypes. Further analysis of module membership (MM) and gene significance (GS) was conducted to identify hub genes. To validate the immune-related modules identified by WGCNA, the CIBERSORT algorithm was used to perform immune infiltration analysis by combining a mouse-derived immune cell signature matrix and the sample gene expression matrix. A linear kernel nu-support vector regression (nu-SVR) was used, and nu values of 0.25, 0.5, and 0.75 were tested to select the best model. Quantile normalization was enabled (QN = TRUE). The number of permutation tests was set to 1000 (perm = 1000) for calculating the *p*-value. The estimated proportions of immune cell types for each sample were used for subsequent analysis and visualization.

### 2.9. Immunofluorescence Staining Was Performed to Assess the Level of CD4^+^ T Cell Infiltration in Liver Tissues

Paraffin sections were dewaxed in xylene and rehydrated through a graded ethanol series to water, followed by antigen retrieval to fully expose antigenic epitopes. After washing, the sections were treated with 3% H_2_O_2_ at room temperature to inhibit endogenous peroxidase, and subsequently blocked with 3% BSA to prevent nonspecific binding. For the first round of staining, CD4 primary antibody (Abcam, ab183685, diluted as per the provided instructions, Cambridge, UK) was added dropwise and incubated overnight at 4 °C. Horseradish peroxidase (HRP)-labeled secondary antibody was added the next day and incubated for 1 h at room temperature for color development. After color development was completed, antigen retrieval was used to elute the first-round antibody to eliminate residual antibody signals. For the second round of staining, CD44 primary antibody (Abcam, ab189524, diluted at the ratio recommended in the instructions) was added dropwise and incubated overnight at 4 °C. The next day, HRP-labeled secondary antibody was added dropwise in the same manner, incubated at room temperature, and TSA color development was performed using a different fluorescence channel from the first round. After all rounds were completed, the cell nuclei were counterstained with DAPI, and the slides were mounted with an anti-fluorescence quenching mounting medium. Images were scanned and captured under a fluorescence microscope with a 20× objective lens. Cells with colocalization of CD4 and CD44 signals are identified as CD4^+^ memory T cells. Image quantitative analysis was performed using ImageJ software.

### 2.10. miR-340 Mimic Transfection in AML12 Cells

The experimental setup included a Ctrl group and a miR-340 mimic group. Twenty-four hours before transfection, AML12 cells were seeded at 5 × 10^5^ cells/well in six-well plates to reach 70–80% confluence at the time of transfection. Before transfection, the medium in each well was replaced with 2 mL of pre-warmed complete medium. A solution of 100 pmol mimics in 125 μL of antibiotic-free, serum-free DMEM was prepared, then 4 μL of Lipo8000 TM transfection reagent (Beyotime, Cat.No. C0533, Shanghai, China) was added and mixed gently. After preparation, the mixture was placed at room temperature and added to each well within 6 h, and the cells were cultured for 48 h before being washed once and collected in PBS.

### 2.11. Isolation, Enrichment, and Electroporation of Primary CD4^+^ T Cells with miR-340 Mimics

Aseptically remove the spleen from 6–8-week-old SPF-grade C57BL/6 mice and gently grind it on a 70 μm cell strainer while rinsing with pre-chilled sterile PBS buffer (containing 2% FBS and 2 mM EDTA) to collect a single-cell suspension. Centrifuge at 500× *g* for 5 min and discard the supernatant. Add ACK red blood cell lysis buffer to the cell pellet and incubate at room temperature for 5 min to remove red blood cells. Terminate lysis by adding PBS containing 10% FBS; then centrifuge, discard the supernatant, and resuspend the cells in PBS. Filter the suspension again through a 70 μm strainer before subsequent CD4^+^ T cell enrichment. Naive CD4^+^ T cells were isolated using the MagniSort^TM^ Mouse CD4 Naive T cell Enrichment Kit (Thermo Fisher, 8804-6824-74, Waltham, MA, USA) from eBioscience according to the instructions. Subsequently, miR-340 mimics or negative control mimics were transfected using a Lonza 4D-Nucleofector^TM^ electroporator (Lonza Cologne GmbH, Köln, Germany). After 24 h of transfection, cells were collected and used for RT-qPCR.

### 2.12. qPCR Detection of miR-340-5p and Psat1 Expression in Liver Tissues and Psat1 Expression in Transfected Cells

After grinding the liver tissue in liquid nitrogen, add 1 mL of TRIzol reagent and vortex thoroughly to mix, then let stand at room temperature for 3–5 min. Add 1/5 volume of chloroform, vortex vigorously for 15 s, let stand at room temperature for 5 min, then centrifuge at 4 °C and 12,000 rpm for 15 min, and carefully aspirate the upper aqueous phase into a new centrifuge tube. Subsequently, an equal volume of isopropanol was added, and the mixture was allowed to stand at room temperature for 20–30 min, followed by centrifugation at 4 °C and 12,000 rpm for 15 min, and the supernatant was discarded. Wash the RNA pellet with 75% ethanol, centrifuge at 8000 rpm for 10 min at 4 °C, discard the ethanol, air-dry at room temperature, and dissolve in an appropriate amount of nuclease-free water to obtain total RNA. According to the manufacturer’s instructions, TaKaRa’s reverse transcription kit (Takara Bio, Cat. No. RR047A, Shiga, Japan) was used to transcribe RNA into cDNA. The relative expression levels of Psat1 and miR-340-5p were detected using a qPCR kit (TransGen Biotech, Cat. No. AQ601-02-V2, Beijing, China), with GAPDH as the internal reference for mRNA and U6 as the internal reference for miRNA. Similarly, AML12 cells at 48 h post-transfection and primary CD4^+^ T cells at 24 h post-transfection underwent the removal of culture medium, a single wash with PBS, and the addition of an appropriate volume of TRIzol. Total RNA was then extracted, followed by reverse transcription and quantitative PCR (qPCR) using the same procedures described above to assess changes in Psat1 mRNA expression separately. Relative gene expression levels were calculated using the 2^−ΔΔCt^ method. The primer sequences used for qPCR are listed in Table 3.

### 2.13. Detection of Psat1 Protein Expression in Liver Tissues by Western Blotting

A lysis buffer was prepared using RIPA, a protease/phosphatase inhibitor cocktail, and PMSF in a ratio of 1000:20:10. Liver tissues of comparable size were lysed in 1000 µL of the buffer on ice for 30 min. Following centrifugation at 12,000 rpm for 10 min, the supernatants were collected, and protein concentrations were measured using a BCA kit. Protein lysates were mixed with loading buffer (4:1), denatured at 95 °C for 10 min, and resolved by SDS-PAGE. The proteins were subsequently transferred onto PVDF membranes at 300 mA for a duration of 0.5 to 2 h, depending on the size of the target protein. The membranes were briefly rinsed with TBST, blocked with 5% non-fat milk for 2 h at room temperature, and incubated with primary antibodies (1:2000) overnight at 4 °C. After three washes with TBST (10 min each at 80 rpm), the membranes were incubated with secondary antibodies (1:5000) for 1 h at room temperature, followed by three additional washes with TBST (10 min each at 60 rpm). Protein signals were detected using an ECL substrate, and images were acquired with optimized exposure settings.

### 2.14. Immunofluorescence Detection of Hepatic Memory CD4^+^ T Cells

Following deparaffinization to water, the sections were subjected to antigen retrieval in a solution at medium-high heat for 30 min. After natural cooling, the sections were washed with PBS. To inhibit endogenous peroxidase activity, the sections were incubated with 3% hydrogen peroxide at room temperature in the dark for 10 min, followed by washing with PBS and blocking with 3% BSA at room temperature for 30 min. The primary antibody working solution was then applied and incubated overnight at 4 °C. The next day, the sections were allowed to reach room temperature and washed with PBS. Subsequently, the HRP-conjugated secondary antibody was added and incubated at room temperature for 30 min, followed by washing with PBS and incubation with TSA working solution at room temperature in the dark for 10 min. The sections were then heated again in the antigen retrieval solution at medium-high heat for 30 min to elute the antibodies, followed by natural cooling and washing with PBS. These steps were repeated for a second round of labeling. DAPI was utilized to stain the cell nuclei, and images were captured using a 20× objective lens. Quantitative analysis of the fluorescence signal was conducted by ImageJ software.

### 2.15. Statistical Analysis

Data are presented as mean ± standard error of the mean (SEM). The Shapiro–Wilk test was used to assess data normality, and the F-test was used to assess homogeneity of variance (only performed when data met a normal distribution). When comparing two independent samples, if the data satisfy a normal distribution and homogeneity of variance, the unpaired Student’s *t*-test is used. If a normal distribution is satisfied but the variances are unequal, the *t*-test with Welch’s correction is used; if a normal distribution is not satisfied, the Mann–Whitney test is used. Repeated-measures data were statistically analyzed using repeated-measures analysis of variance (ANOVA). All tests were two-tailed; *p* < 0.05 was considered statistically significant (* *p* < 0.05, ** *p* < 0.01, *** *p* < 0.001, **** *p* < 0.0001). All statistical analyses were performed using GraphPad Prism software (version 10.1.2).

## 3. Results

### 3.1. The Combination of a High-Fat and High-Sugar Diet and a High-Temperature and High-Humidity Environment Induces Abnormalities in General Condition and Behavior in CHB Mice

**Figure 2:**
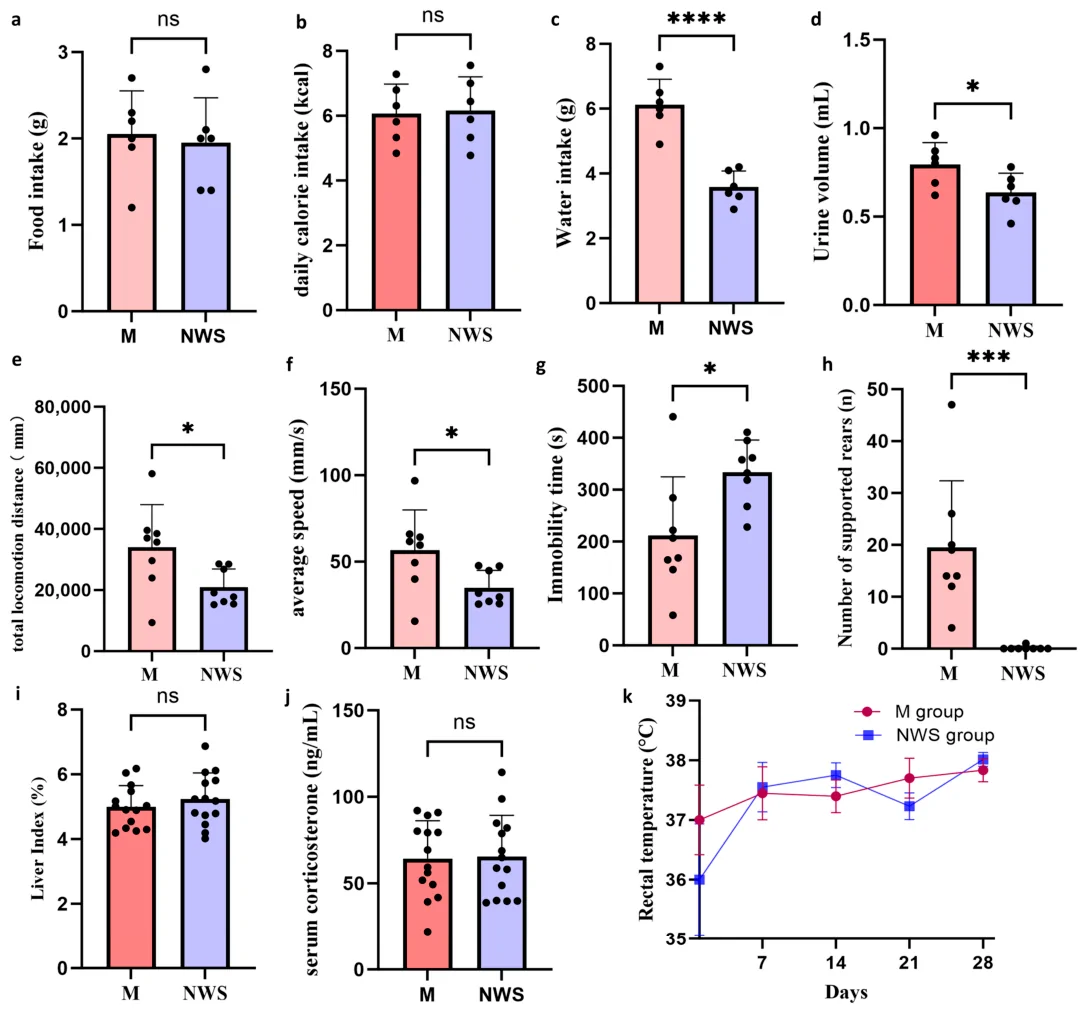
Comparison of general status and behavior-related indicators between two groups of mice. (**a**) Food intake. (**b**) Daily calorie intake. (**c**) Water intake. (**d**) Urine characteristics. (**e**) Total locomotion distance. (**f**) Average speed. (**g**) Immobility time. (**h**) Number of supported rears. (**i**) Liver index. (**j**) Serum corticosterone. (**k**) Rectal temperature. ns indicates *p* > 0.05, * *p* < 0.05, *** *p* < 0.001, **** *p* < 0.0001, compared with the M group.

To evaluate the effects of a high-fat and high-sugar diet combined with a high-temperature and high-humidity environment on the overall physiological state of CHB mice, we conducted comprehensive observations in terms of food intake and energy intake, water intake and excretion, body temperature changes, and behavioral activity. The metabolic cage monitoring results showed that, compared with the M group, the NWS group exhibited reduced 24-h urine volume (*p* < 0.05), and the urine color changed from clear light yellow to darker yellow (Figure 2d). Meanwhile, water intake was significantly reduced in the NWS group (*p* < 0.0001, Figure 2c), whereas no significant differences were observed in daily food intake or energy intake between the two groups (*p* > 0.05, Figure 2a,b). The above results suggest that the intervention primarily affects water balance rather than total energy intake. Continuous 4-week temperature monitoring showed that the rectal temperatures of the two groups of mice were generally maintained within a relatively stable range, but the NWS group exhibited a more pronounced trend of temperature fluctuation during the observation period (Figure 2k). Further open field behavioral tests revealed that, compared to the M group, the total locomotion distance of the mice in the NWS group was significantly reduced (*p* < 0.05). Additionally, the average speed decreased (*p* < 0.05), the number of support rears diminished (*p* < 0.001), and the immobility time increased (*p* < 0.05) (Figure 2e–h). These findings suggest a decline in autonomic activity levels and a reduction in exploratory behavior among the NWS group. No significant difference was observed in the liver weight/body weight ratio between the NWS group and the M group (*p* > 0.05, Figure 2i), suggesting that the high-fat and high-sugar diet did not cause a significant increase in relative liver weight under the conditions of this experiment. Additionally, the levels of serum corticosterone showed no significant variation between the M and NWS groups (*p* > 0.05, Figure 2j), implying that systemic glucocorticoid baselines might be similar and that corticosterone levels are probably not responsible for the differences observed between the two groups. These findings indicate that the combined intervention mainly induces alterations in water balance and behavioral abnormalities, while having no significant effect on energy intake or systemic corticosterone levels.

### 3.2. High-Fat and High-Sugar Diet Combined with High-Temperature and High-Humidity Environment Aggravates Liver Injury and HBV Infection Phenotype

**Figure 3:**
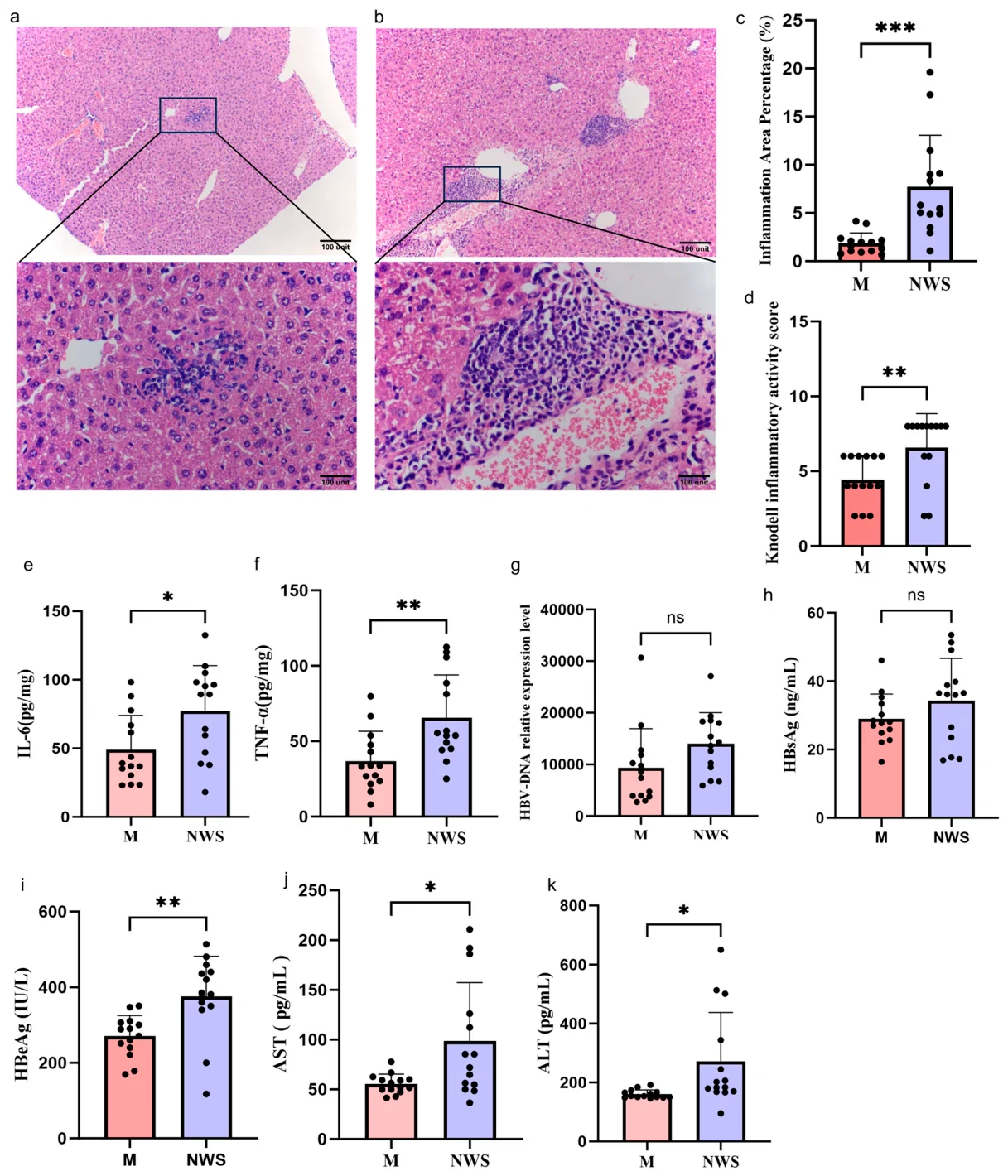
Evaluation of the CHB mouse model. (**a**,**b**) Histopathological observation of mouse liver tissue stained with HE (scale bar: 100 μm). (**c**) Inflammation area percentage. (**d**) Knodell inflammatory activity score. (**e**,**f**) Expression levels of IL-6 in liver tissue. (**g**) Relative expression level of HBV-DNA in liver tissue. (**h**,**i**) Expression levels of HBsAg and HBeAg in serum. (**j**,**k**) Expression levels of AST and ALT in serum. ns indicates *p* > 0.05, * *p* < 0.05, ** *p* < 0.01, *** *p* < 0.001, compared with the M group.

After 4 weeks of modeling, both the M group and the NWS group successfully established the CHB model. Further analysis of HBV-related virological indicators between the two groups showed that while intrahepatic HBV expression levels were slightly higher in the NWS group than in the M group, the difference was not statistically significant (Figure 3g; *p* > 0.05). According to peripheral blood testing (Figure 3h,i), HBsAg levels in the NWS group exceeded those in the M group without reaching statistical significance (*p* > 0.05), but HBeAg levels were significantly higher than those found in the M group (*p* < 0.01). Meanwhile, peripheral blood ALT and AST levels in the NWS group were higher than those in the M group, and the differences were statistically significant (*p* < 0.05; Figure 3j,k), suggesting that exposure to a high-fat, high-sugar diet combined with a high-temperature, high-humidity environment further aggravated hepatocellular injury in CHB mice. H&E staining showed that the hepatic lobule structure in group M was basically intact, with hepatocytes arranged radially; only focal inflammatory cell infiltration and mild hydropic degeneration were observed, with no obvious steatosis (Figure 3a). In contrast, the NWS group exhibited more pronounced hepatic pathological changes, with extensive inflammatory cell infiltration visible in the portal area and around the central/hepatic veins, accompanied by significant microvesicular steatosis, with the cytoplasm filled with uniform small lipid droplets (Figure 3b). Histological quantitative analysis further confirmed (Figure 3c,d) that the area of inflammatory cell infiltration in liver tissue (*p* < 0.001) and the inflammatory score (*p* < 0.01) in the NWS group were significantly higher than those in the M group. Consistent with the aforementioned pathological changes, the levels of IL-6 (*p* < 0.05) and TNF-α (*p* < 0.01) in the liver tissue of the NWS group were significantly higher than those in the M group, with statistically significant differences (Figure 3e,f), indicating a further enhancement of local inflammatory responses in the liver. In summary, combined exposure exacerbated liver inflammatory infiltration and steatosis in the CHB model mice, accompanied by elevated peripheral blood ALT, AST, and liver tissue levels of IL-6 and TNF-α. Meanwhile, HBV-related virological indicators also showed varying degrees of increase.

### 3.3. Identification of Differentially Expressed miRNAs, mRNAs, and Proteins in Liver Tissues

**Figure 4:**
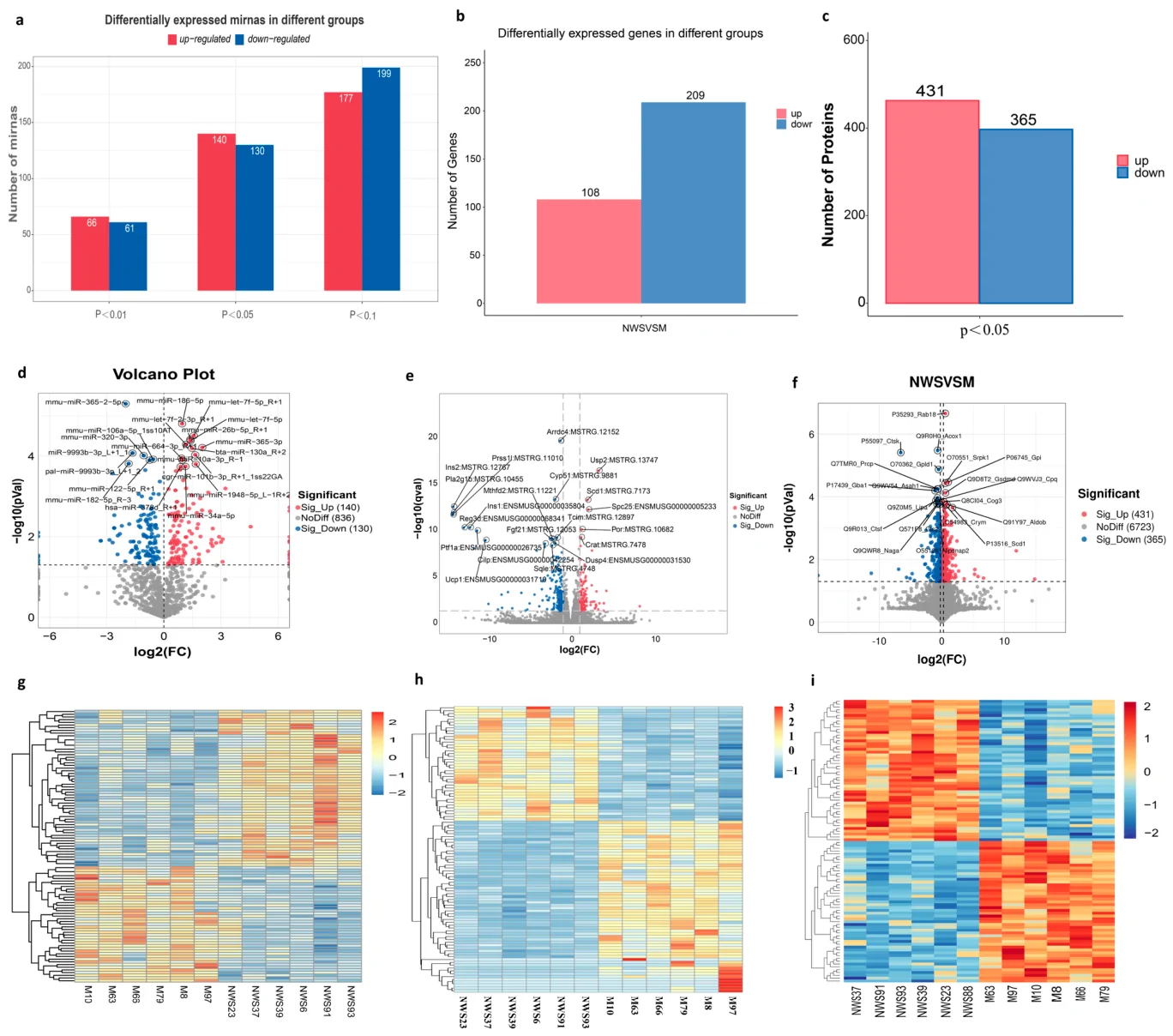
Analysis of differentially expressed genes and overall protein characteristics in the liver of CHB mice under a high-fat, high-sugar diet combined with a high-temperature, high-humidity environment. (**a**) Statistical chart of differentially expressed miRNAs. (**b**) Statistical chart of differentially expressed mRNAs. (**c**) Statistical chart of differentially expressed proteins. (**d**) Volcano plot of differentially expressed miRNAs. (**e**) Volcano plot of differentially expressed mRNAs. (**f**) Volcano plot of differentially expressed proteins. (**g**) Clustering heat map of differentially expressed miRNAs. (**h**) Clustering heat map of differentially expressed mRNAs. (**i**) Clustering heat map of differentially expressed proteins.

To explore the potential mechanisms and molecular targets of a high-fat, high-sugar diet combined with a high-temperature, high-humidity environment in the pathogenesis of CHB, this study employed whole-transcriptome and proteome sequencing for analysis (Figure 4). To evaluate the impact of different significance thresholds on the identification of differentially expressed miRNAs, we set multiple thresholds for comparative analysis (Figure 4a). At *p* < 0.01, 127 differentially expressed miRNAs were identified, with 66 upregulated and 61 downregulated. At *p* < 0.05, 270 differentially expressed miRNAs were identified, including 140 upregulated and 130 downregulated. At *p* < 0.1, 376 differentially expressed miRNAs were identified, with 177 upregulated and 199 downregulated. The volcano plot (Figure 4d) shows that while the majority of miRNAs showed no differential expression (NoDiff), several significantly upregulated (Sig_Up) and significantly downregulated (Sig_Down) miRNAs were evident in the flanking regions, indicating a clear distinction in miRNA expression between the two groups. Heat map analysis (Figure 4g) demonstrated that the two groups were clearly separated, with high consistency among biological replicates within each group and significant differences between groups.

At the mRNA level, a total of 317 differentially expressed genes were identified, including 209 downregulated and 108 upregulated genes (Figure 4b). The volcano plot (Figure 4e) highlighted several differentially expressed genes, including downregulated genes such as *Arrdc4, Ins2, Prss1,* and *Reg3,* as well as upregulated genes such as *Usp2, Scd1, Spc25*, and *Por.* The heat map (Figure 4h) showed clear separation between the two groups and high intra-group reproducibility. Proteomic analysis (Figure 4c) identified a total of 796 significantly differentially expressed proteins, of which 431 were upregulated and 365 were downregulated. The volcano plot (Figure 4f) revealed a set of significantly upregulated and downregulated proteins. Among the significantly upregulated proteins were Rab18 (P35293), Srpk1 (Q70551), and Ctsf (Q9R013), while the significantly downregulated proteins included Acox1 (Q9R0H0), Ctsk (P55097), and Prop (Q7TMR0). The heat map (Figure 4i) showed that the protein expression profiles differed significantly between the M group and the NWS group, while the expression characteristics of samples within each group were highly consistent. Overall, integrated transcriptomic and proteomic analysis revealed that a high-fat, high-sugar diet combined with a high-temperature, high-humidity environment induced extensive and significant differences in miRNA, mRNA, and protein expression in the CHB model.

### 3.4. Identification of the Brown Module as a Key Functional Regulatory Module Based on WGCNA

**Figure 5:**
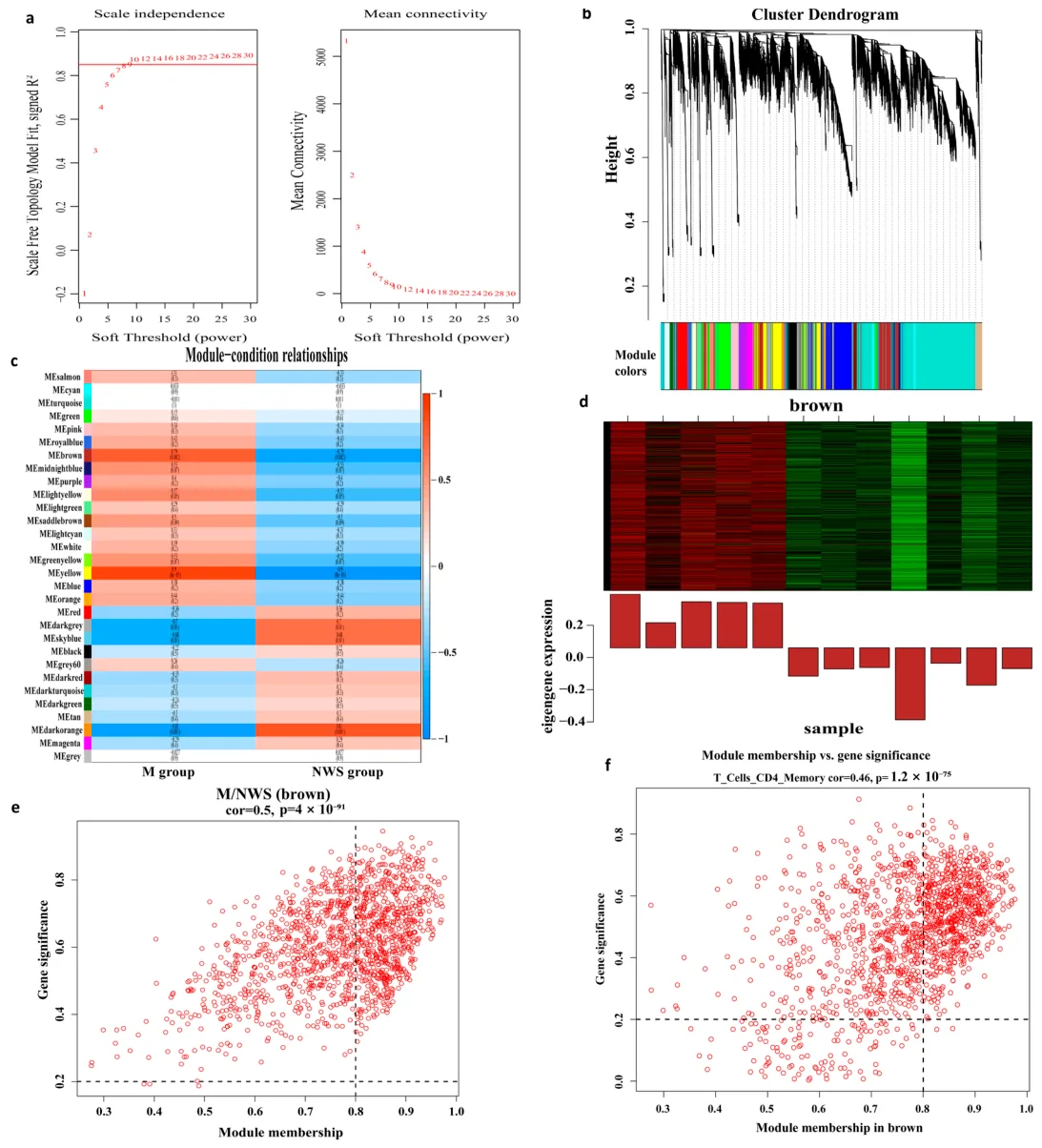
Key gene modules associated with CHB under combined exposure to a high-fat, high-sugar diet and a high-temperature, high-humidity environment, as revealed by WGCNA. (**a**) Determination of the soft-thresholding power (β) for WGCNA. (**b**) Hierarchical clustering dendrogram of genes based on topological overlap (with color-coded annotations below the dendrogram). (**c**) Heatmap of module–trait relationships. (**d**) Module-gene heatmap. (**e**,**f**) Scatter plots of module membership (MM) versus gene significance (GS).

To identify gene modules associated with the CHB phenotype mediated by a high-fat, high-sugar diet combined with a high-temperature, high-humidity environment, this study employed weighted gene co-expression network analysis (WGCNA). We determined the optimal soft-thresholding power to be 9 (Figure 5a), based on a scale-free topology fit index > 0.85 and moderate mean connectivity. Hierarchical clustering identified multiple gene modules, which were visualized using dendrograms and color coding (Figure 5b). A total of 30 gene co-expression modules were constructed using WGCNA (Table 4), with marked differences in gene numbers among modules. Among these modules, the turquoise module contained the highest number of genes (5096), followed by the blue (1772), brown (1427), and yellow (1283) modules. The green (1052) and red (552) modules fell within the middle range, while the grey (9) and saddlebrown (36) modules contained only a very small number of genes. The remaining modules exhibited gene counts generally ranging between 150 and 400. The module–trait relationship heatmap (Figure 5c) indicated that, compared with the M group, the brown module (|r| = 0.79, *p* = 0.002) and the yellow module (|r| = 0.90, *p* = 8 × 10^−5^) were strongly negatively correlated with the NWS group, whereas the dark orange module (|r| = 0.81, *p* = 0.001) was positively correlated. The module-gene heatmap (Figure 5d) further demonstrated that the expression profiles of the brown module in the M and NWS groups showed a distinct separation trend, suggesting that the expression profile of this module was highly heterogeneous between the two groups. Additionally, the scatter plot of module membership (MM) and gene significance (GS) (Figure 5e) confirmed a significant correlation for the brown module (cor = 0.51, *p* = 4 × 10^−91^). We further analyzed the genes within the brown module by plotting module membership against gene significance (Figure 5f). The results demonstrated a strong positive correlation between module membership and gene significance for CD4^+^ T-cell-related genes in the brown module (cor = 0.46, *p* = 1.2 × 10^−75^). Consequently, we identified memory CD4^+^ T cells as a key immune cell population associated with this module.

### 3.5. CIBERSORT Indicated That a Combination of a High-Fat, High-Sugar Diet and Exposure to High Temperature and Humidity Decreased the Infiltration of Liver CD4^+^ Memory T Cells in Mice with Chronic HBV Infection

**Figure 6:**
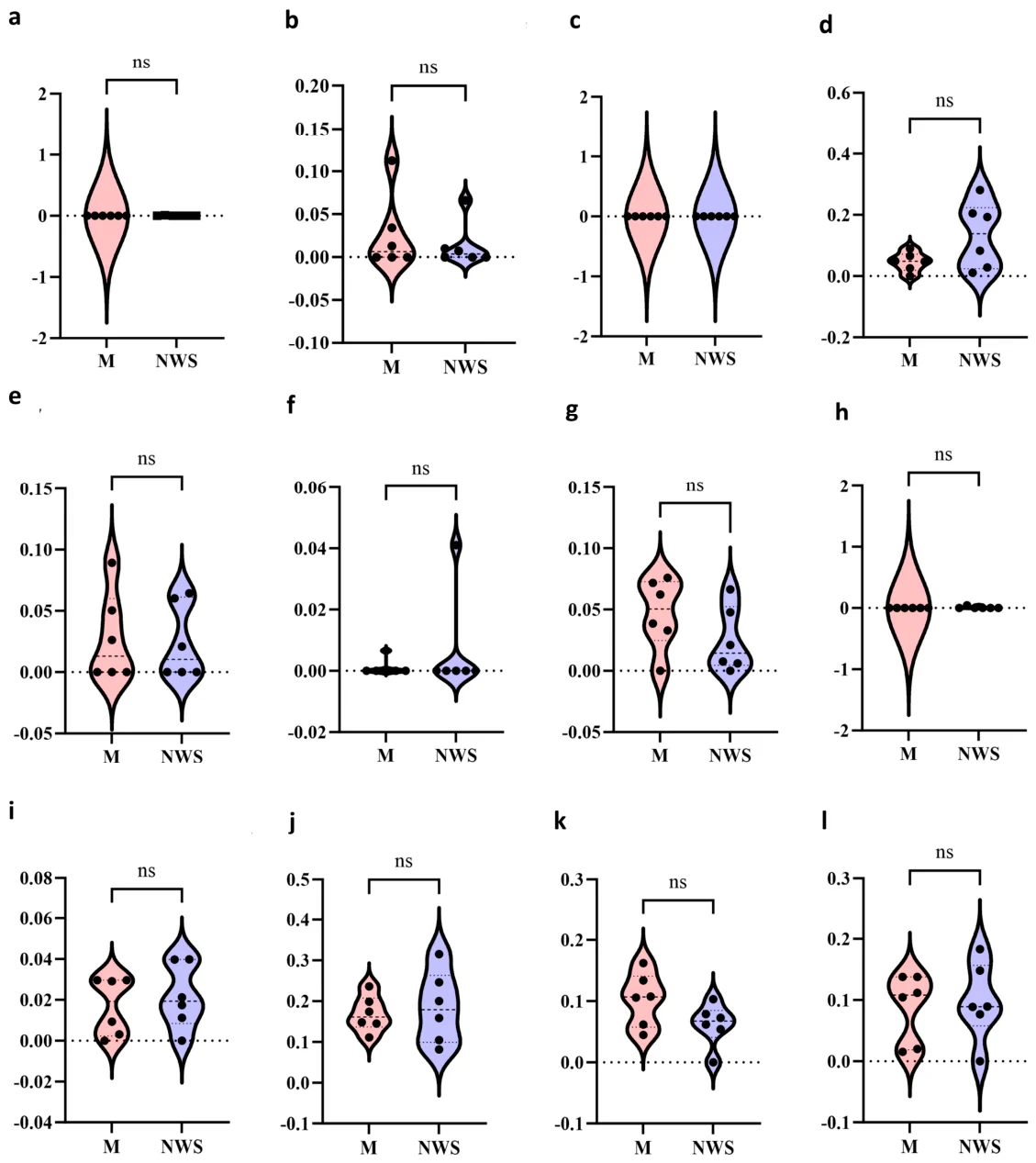
Violin plots showing the infiltration proportions of immune cell subsets in the liver. (**a**) memory B cells; (**b**) CD4^+^ naive T cells; (**c**) mast cells; (**d**) M0 macrophages; (**e**) memory CD8^+^ T cells; (**f**) follicular CD4^+^ T cells; (**g**) activated NK cells; (**h**) Th2 cells (T helper 2 cells); (**i**) γδ T cells; (**j**) monocytes; (**k**) Treg cells (regulatory T cells); (**l**) naive B cells. ns indicates *p* > 0.05, compared with the M group.

**Figure 7:**
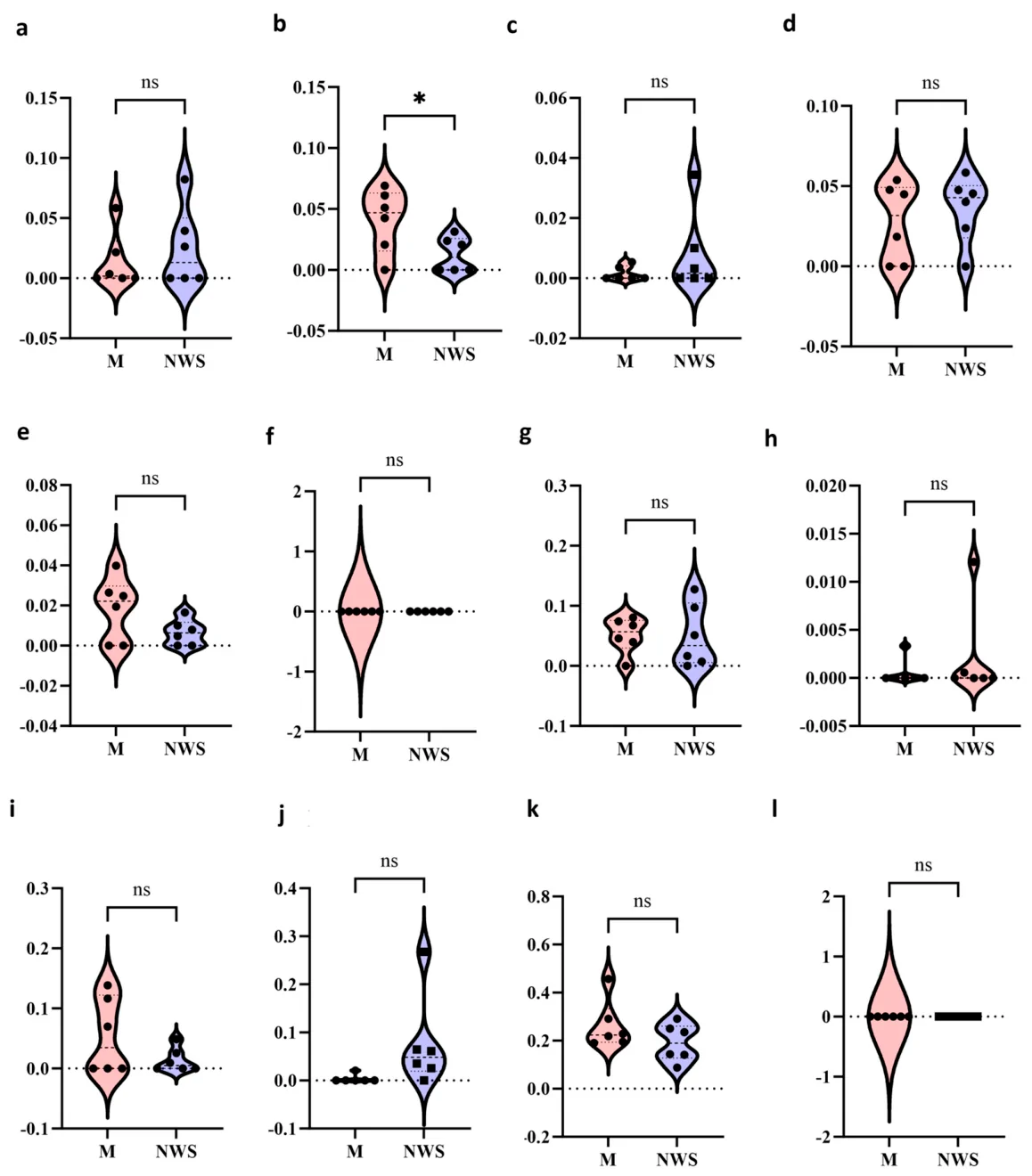
Analysis of immune cell subset infiltration proportions in the liver via violin plots. (**a**) naive CD8^+^ T cells; (**b**) memory CD4^+^ T cells; (**c**) activated CD8^+^ T cells; (**d**) plasma cells; (**e**) Th17 cells; (**f**) Th1 cells; (**g**) immature dendritic cells; (**h**) resting NK cells; (**i**) M2 macrophages; (**j**) neutrophils; (**k**) M1 macrophages; (**l**) eosinophils. ns indicates *p* > 0.05, * *p* < 0.05, compared with the M group.

To further elucidate and validate the characteristics of the immune microenvironment, we employed the CIBERSORT algorithm to assess the immune cell infiltration profiles of liver tissues from the M group and the NWS group. The proportions of immune cell subpopulations and intergroup differences were visualized using violin plots (Figure 6 and Figure 7). The analysis revealed that the immune cell composition in liver tissue of the NWS group predominantly showed two main trends. The cell subpopulations with upward trends were mainly classified as innate immunity and naive lymphocyte populations. Specifically, these included naive B cells (Figure 6l), immature dendritic cells (Figure 7g), γδ T cells (Figure 6i), M0 macrophages (Figure 6d), neutrophils (Figure 7i), and activated CD8^+^ T cells (Figure 7c). In contrast, the cell populations with downward trends were mainly concentrated in adaptive immune regulatory and effector cell subsets, including Th17 cells (Figure 7e), Treg cells (Figure 6k), M2 macrophages (Figure 7i), M1 macrophages (Figure 7k), memory CD4^+^ T cells (Figure 7b), and CD4^+^ naive T cells (Figure 6b). The remaining immune cell subsets not classified above did not show significant differences in distribution between the two groups. While some cell subsets showed notable upregulation or downregulation trends in the NWS group, differences in immune cell infiltration between the two groups were not statistically significant for most subsets. Importantly, memory CD4^+^ T cells (Figure 6b) showed a significant downward trend (*p* < 0.005), suggesting that the reduction in this subset may be a key feature of impaired adaptive immune responses in the CHB mouse model, particularly under a high-fat, high-sugar diet combined with exposure to a high-temperature, high-humidity environment. In conclusion, the results from the CIBERSORT immune infiltration analysis aligned closely with the trends of key immune cell changes identified in the previous WGCNA. This consistency further underscored the potentially significant role of memory CD4^+^ T cells in the remodeling of the immune microenvironment within the context of a high-fat, high-sugar diet and a high-temperature, high-humidity environment, thus providing a foundation for subsequent elucidation of the immune regulatory mechanisms of this subset.

### 3.6. Multi-Omics Association Analysis Based on WGCNA-Derived Key Modules Reveals the Function of the miR-340-5p/Psat1 Signaling Pathway in Memory CD4^+^ T Cells

To further filter core genes associated with memory CD4^+^ T cell function, this study conducted a multi-level intersection analysis. Initially, all genes (*n* = 1427) in the brown module that were significantly associated with the phenotype were extracted from the WGCNA and intersected with 317 differentially expressed mRNAs obtained from transcriptomic differential analysis. A total of 51 candidate genes were identified, which showed significant differential expression characteristics and were located within key co-expression modules. Subsequently, an absolute correlation threshold of |r| ≥ 0.5 for memory CD4^+^ T cells was applied, and the aforementioned candidate genes were further intersected with a set of 713 genes significantly associated with this immune cell type, ultimately identifying 36 core mRNAs closely related to memory CD4^+^ T cells (Table 5). Among these, 13 were annotated genes, including *Chic1, Dzank1, Trim45, Cd209b, Nabp1, Adnp2, Ptges, Psat1, Asns, Rragb, Mthfr, Prom1,* and *Gadl1*, while the remaining 23 were unannotated transcripts.

**Figure 8:**
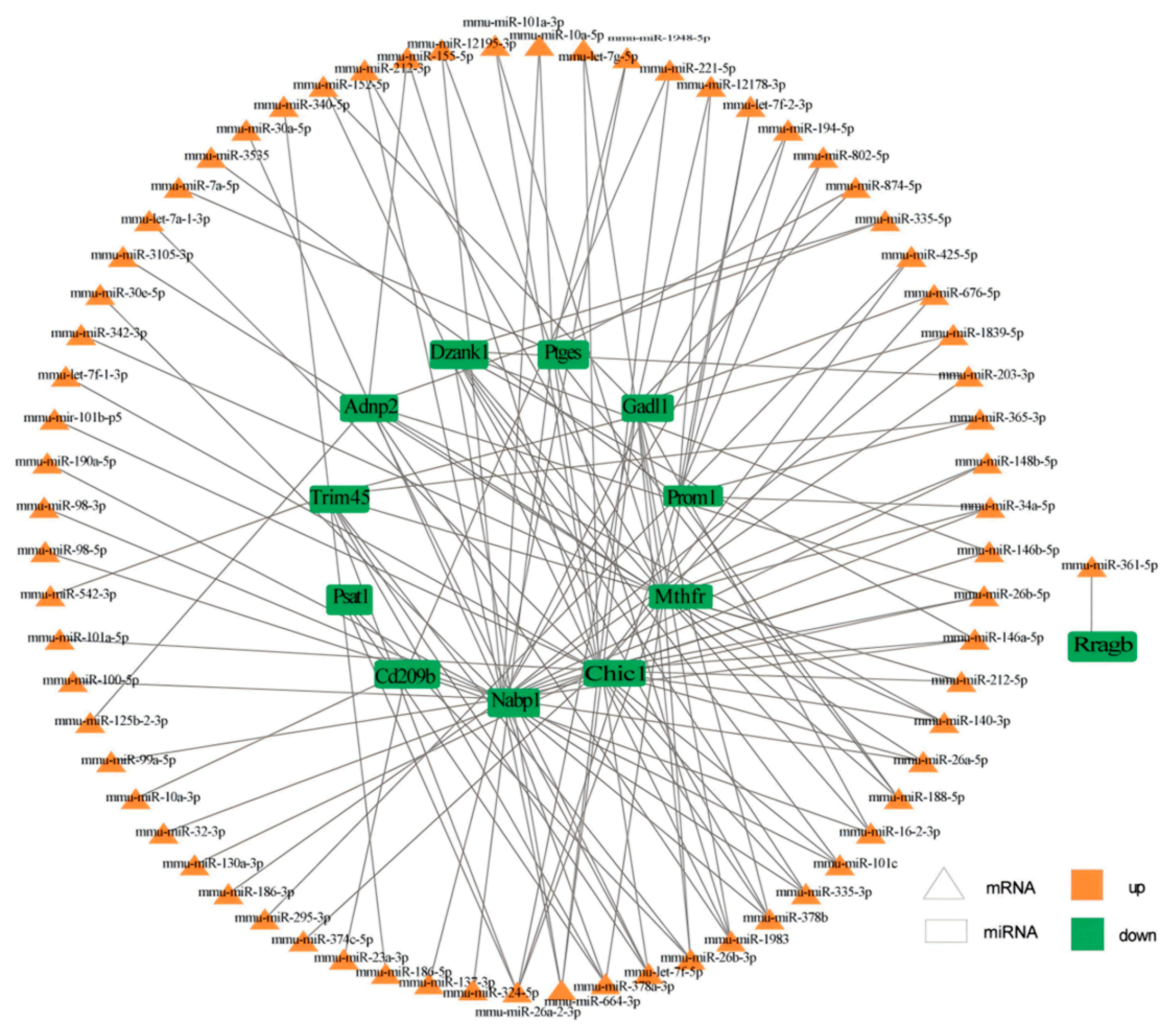
Core miRNA-mRNA regulatory network analyzed by Cytoscape software. In this network, miRNAs act as regulatory hubs and mRNAs as effector targets, establishing a bipartite post-transcriptional regulatory framework that facilitates the identification of critical miRNAs involved in modulating memory CD4^+^ T cell function.

In this study, we identified 13 annotated mRNAs and differentially expressed miRNAs through screening and conducted an integrated analysis combining target gene prediction and expression correlation. This analysis revealed a total of 750 miRNA-mRNA interaction pairs with potential regulatory relationships. Further screening, combined with expression trends, yielded 390 pairs of significantly negatively correlated upregulated miRNA-downregulated mRNA interaction relationships. These findings suggest that these miRNAs regulate target gene expression via post-transcriptional repression. In the negative correlation interaction network, notable differences were found in the number of potential regulatory miRNAs associated with various genes. Specifically, *Chic1* was associated with 35 miRNAs, *Dzank1* with 30, *Trim45* with 12, *Cd209b* with 7, *Nabp1* with 133, *Adnp2* with 11, *Ptges* with 12, *Psat1* with 8, *Rragb* with 3, *Mthfr* with 41, *Prom1* with 69, and *Gadl1* with 29. Considering biological relevance, this study further focused on mouse-derived miRNA-mRNA interaction pairs, ultimately identifying 338 regulatory relationships for subsequent analysis. To visually represent these regulatory relationships, we constructed a miRNA-mRNA regulatory network using Cytoscape (version: 3.10.4) (Figure 8). This network clearly illustrates the complex regulatory interactions between differentially expressed miRNAs and their candidate target genes, providing a crucial foundation for further screening of key miRNAs and core regulatory axes involved in the regulation of memory CD4^+^ T cell function.

Correlation analysis was conducted between the annotated genes and proteomic data, resulting in the identification of three gene-protein pairs with consistent expression patterns: Prom1/O54990, Mthfr/Q9WU20 and Psat1/Q99K85. The gene Prom1 encodes the cell surface glycoprotein Prominin-1 (CD133), corresponding to UniProt entry O54990. Functional annotation revealed that this protein is primarily localized to membrane structures, including the apical plasma membrane, brush border, and microvilli, and is involved in biological processes associated with the extracellular environment. KEGG analysis indicated its involvement in the “transcriptional misregulation in cancer” pathway, suggesting that Prom1 may play a role in cell fate maintenance and stem cell-related regulatory processes. Previous studies have demonstrated that upon induction by miR-146a and miR-150, CD133^+^ hematopoietic progenitor cells can be directed to differentiate into CD4^+^ T cells, supporting a potential role for Prom1 in regulating T-cell development and maintaining immune homeostasis [37].

The protein Q9WU20 corresponding to Mthfr is mainly localized in the cytoplasm and is an important regulatory enzyme in folate-mediated one-carbon metabolism. Pathway annotation shows that Mthfr is involved in folate one-carbon metabolism, folate metabolism, and antifolate drug resistance-related pathways. Mthfr catalyzes the conversion of 5,10-methylenetetrahydrofolate to 5-methyltetrahydrofolate, serving as a key metabolic branch point connecting the folate cycle and the methionine cycle [38]. Decreased MTHFR activity can reduce the production of 5-methyltetrahydrofolate, thereby affecting homocysteine remethylation, the methionine cycle, and the SAM/SAH balance, thus altering the methylation capacity of cells [39,40]. Existing pan-cancer analyses based on the xCell algorithm have shown that Mthfr expression is positively correlated with the infiltration levels of CD4^+^ T cells, CD8^+^ T cells, and naive CD4^+^ T cells [41]. Meanwhile, folate is particularly important for T cell proliferation, and its deficiency can lead to a reduction in the number of circulating T cells and a decline in the antigen-responsive capacity of T cells, thereby exacerbating the disease state [42,43]. The above results suggest that Mthfr-mediated metabolic alterations may be associated with T cell immune status. However, their specific regulatory role and causal relationship still require further verification.

Psat1 corresponds to protein Q99K85, which is a key metabolic enzyme in the de novo serine synthesis pathway and participates in the biosynthesis of L-serine [44]. KEGG analysis showed that Psat1 is involved in glycine, serine, and threonine metabolism, as well as cysteine and methionine metabolism, carbon metabolism, and various other metabolic processes. Among them, serine is not only an important raw material for protein synthesis, but can also provide carbon units for purine and thymidylate synthesis through one-carbon metabolism, and its metabolic status is closely related to nucleotide synthesis in rapidly proliferating cells [45]. Existing studies have shown that Psat1 expression is associated with the level of immune cell infiltration in various tumors, including CD8^+^ T cells and CD4^+^ T cells [46]. Consistently, in a colon adenocarcinoma study, Psat1 was screened as a candidate core gene in the PPI and ceRNA networks; the study also observed differences in CD4^+^ memory T cell infiltration between different risk groups [47]. Furthermore, in breast cancer, Treg cells and CD4^+^ memory T cells are significantly enriched in the microenvironment of tumors with high Psat1 expression subtypes [48]. The above studies suggest that Psat1 expression and the serine metabolic status involved may be associated with T cell immune infiltration and immune status. However, the existing evidence mainly comes from tumor research. Whether Psat1 and its mediated alterations in serine metabolism are involved in immune regulation during chronic HBV infection and related liver diseases still lacks direct evidence and awaits further investigation.

Based on the functional analysis of the candidate molecules and a comprehensive review of the literature from CNKI, PubMed, and Web of Science, it was determined that only the targeted regulatory relationship between miR-340-5p and Psat1 has been experimentally validated [49]. Given the critical role of Psat1 in the metabolic reprogramming of memory CD4^+^ T cells, alongside definitive evidence supporting its targeted regulation by miR-340-5p, this study identifies the miR-340-5p/Psat1 pair and further investigates its expression changes in liver tissue.

### 3.7. Correlation of miR-340-5p and Psat1 Expression in Liver Tissues and the Regulatory Effect of miR-340-5p Mimic on Psat1

**Figure 9:**
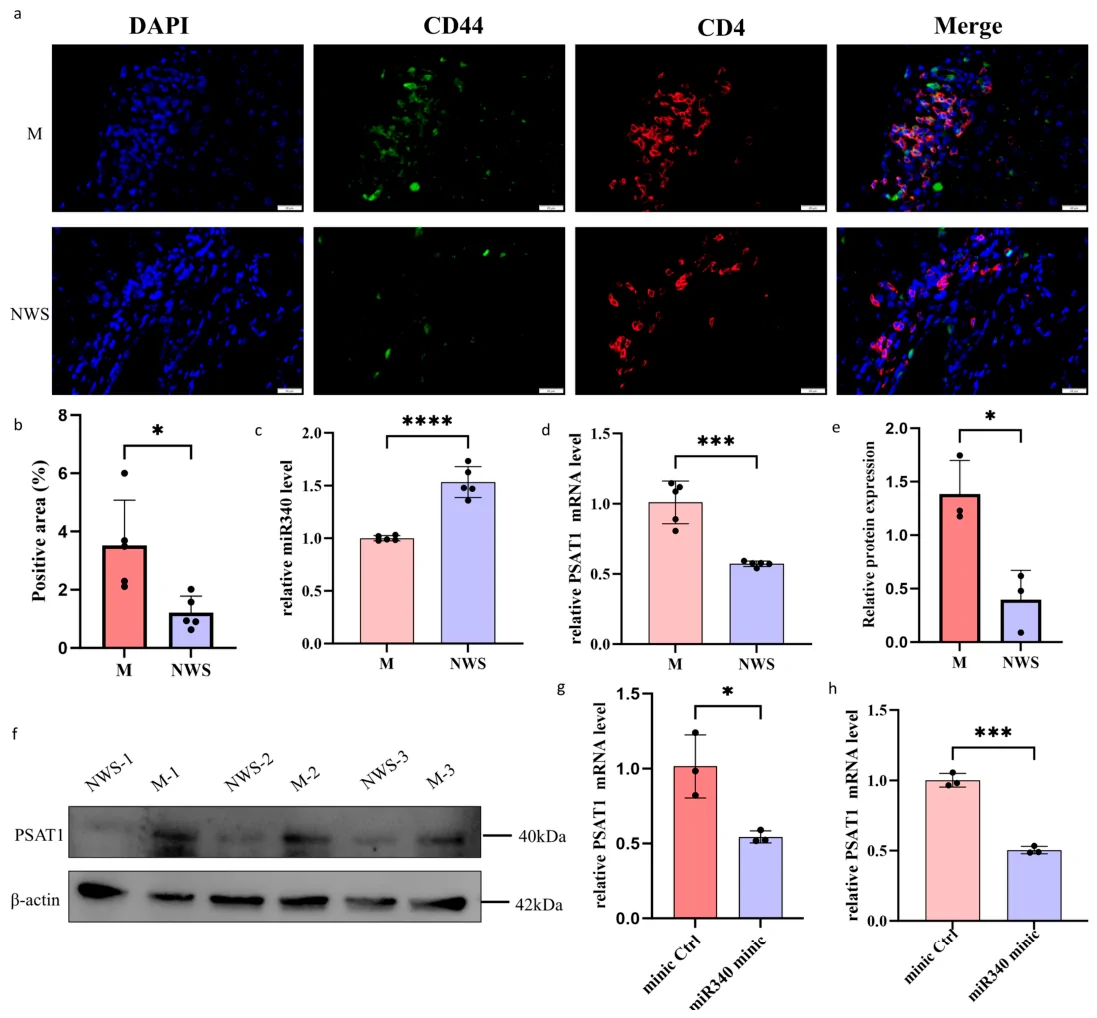
Expression correlation of miR-340-5p and Psat1 in liver tissues and regulation of Psat1 by miR-340-5p mimic in AML12 and CD4^+^ T cells. (**a**) Immunofluorescence staining of memory CD4^+^ T cells in liver tissues (×200; scale bar, 20 μm)). (**b**) Quantification of immunofluorescence. (**c**) miR-340-5p expression levels in liver tissues. (**d**) Psat1 mRNA expression levels in liver tissues. (**e**) Western blot quantification of PSAT1 protein expression in liver tissues. (**f**) Western blot analysis of PSAT1 protein expression in liver tissues. (**g**) Psat1 mRNA expression levels in primary CD4^+^ T cells after transfection with a miR-340-5p mimic. (**h**) Psat1 mRNA expression levels in AML12 cells after transfection with a miR-340-5p mimic. * *p* < 0.05, *** *p* < 0.001, **** *p* < 0.0001, compared with the M group.

This study used immunofluorescence staining to evaluate the effects of a high-fat and high-sugar diet combined with a high-temperature and high-humidity environment on the infiltration of memory CD4^+^ T cells in liver tissue. The results showed (Figure 9a,b) that compared with the M group, the infiltration of memory CD4^+^ T cells in the liver tissue of the NWS group was significantly reduced (*p* < 0.05). This suggests that a high-fat, high-sugar diet and a high-temperature, high-humidity environment may be associated with reduced infiltration of memory CD4^+^ T cells in the liver tissue of HBV-infected mice. Given that previous multi-omics analyses identified miR-340-5p as a potential candidate molecule, we further examined the expression levels of miR-340-5p in liver tissues from each group. The results showed (Figure 9c) that miR-340-5p expression in the NWS group was significantly higher than that in the M group (*p* < 0.0001). Combined with the results of previous multi-omics analyses, it is suggested that elevated miR-340-5p expression may be involved in the regulatory process of reduced CD4^+^ memory T cell infiltration under a high-fat and high-sugar diet combined with a high-temperature and high-humidity environment. To further investigate the possible downstream molecules regulated by miR-340-5p, we used qPCR to detect the mRNA expression level of *Psat1* in liver tissues from each group. As shown in Figure 9d, *Psat1* mRNA expression in the NWS group was significantly lower than that in the M group (*p* < 0.001), exhibiting a trend opposite to the change in miR-340-5p expression, suggesting that elevated miR-340-5p may be associated with the downregulation of Psat1 expression. Western blot analysis was further performed to detect Psat1 protein expression. The results showed that Psat1 protein levels in the NWS group were also significantly decreased (*p* < 0.05), consistent with the changes in *Psat1* mRNA expression (Figure 9e,f). See Supplementary Materials for the densitometric analysis (Table S1), uncropped Western blots (Figure S1), and original images for Figure 9 (File S1). To further verify the regulatory effect of miR-340-5p on *Psat1* expression, primary CD4^+^ T cells and AML12 cells were transfected with a miR-340-5p mimic or a negative control. The experimental results of primary CD4^+^ T cells showed (Figure 9g) that Psat1 expression was significantly reduced after miR-340-5p overexpression (*p* < 0.05). Consistent with the above results, miR-340-5p overexpression also significantly reduced Psat1 mRNA expression in AML12 cells (*p* < 0.001; Figure 9h). The above results further support the existence of a negative regulatory relationship between miR-340-5p and Psat1.

## 4. Discussion

This study employed an efficient rcccDNA/Ad transfection technique to stably deliver HBV genes into hepatocytes, successfully constructing an *Alb-Cre* mouse model of CHB. On this basis, a CHB model reflecting the dual intervention of dietary and environmental factors was established by combining a high-fat, high-sugar diet and a high-temperature, high-humidity environment. This model can reliably reproduce the key pathological features of the coexistence of persistent HBV replication and host immune responses, and effectively simulate the disease progression in which persistent viral presence and chronic liver inflammation coexist over the long term in patients with chronic hepatitis B [34]. Compared with the traditional single-factor model, this two-factor model can more accurately reflect the changes in the local hepatic immune microenvironment of CHB under the combined effects of dietary metabolic abnormalities and environment.

The experimental results indicate that a high-fat, high-sugar diet and a high-temperature, high-humidity environment can significantly aggravate liver injury in CHB model mice. Histopathological analysis showed aggravated hepatocellular steatosis and an expanded range of inflammatory cell infiltration; meanwhile, serum ALT and AST levels were significantly elevated, and the expression of pro-inflammatory cytokines IL-6 and TNF-α in liver tissue was markedly increased. The above results indicate a deterioration of liver function and an intensified inflammatory response. In conclusion, combined exposure to a high-fat, high-sugar diet and a high-temperature, high-humidity environment aggravated hepatic pathological injury in CHB, and this effect may be closely associated with disturbances in the local hepatic immune microenvironment. It is worth noting that although there was no significant difference in HBV expression levels between the NWS group and the M group (*p* > 0.05), the NWS group exhibited more severe overall liver tissue damage and a stronger inflammatory response. These results indicate that even under conditions of relatively stable viral load, combined stimulation by dietary and environmental factors can still significantly exacerbate liver injury in CHB. It also suggests that alterations in the host immune microenvironment, rather than mere changes in viral replication, may be a key mechanism driving disease progression.

Existing studies suggest that miR-340-5p is expressed in various liver cell types and is involved in inflammation and fibrosis associated with chronic liver disease. Clinical studies have shown that the expression level of miR-340-5p is significantly upregulated from NC to CHB, and remains at a high level in both disease stages (CHB and LC) [50]. In HBV-related cirrhosis, miR-340-5p may also be involved in the regulation of inflammatory responses, immune cell function, and hepatic stellate cell activation [51]. In normal liver parenchyma and during the transition from non-alcoholic fatty liver disease and non-alcoholic steatohepatitis to the progressive deterioration toward hepatocellular carcinoma, hepatocytes remain one of the important sources of miR-340-5p [50,52]. It is noteworthy that regardless of whether miR-340-5p specifically originates from hepatocytes or immune cells, both can release it into the tissue microenvironment through exosomal or paracrine pathways. Related studies suggest that miR-340 may be involved in CD4^+^ T cell-related immune regulation. For example, in patients with multiple sclerosis, miR-340 is upregulated in CD4^+^ T cell subsets, suggesting that its aberrant expression may be associated with altered functional states of CD4^+^ T cells [53]. In the spleens of tumor-bearing mice, both CD4^+^ T lymphocytes and CD8^+^ T lymphocytes were significantly upregulated in the miR-340 overexpression group [54]. In this study, using a CHB mouse model subjected to a high-fat, high-sugar diet and a high-temperature, high-humidity environment, it was found that miR-340-5p expression in the liver tissue of mice in the NWS group was significantly increased. However, there is still a lack of direct evidence regarding whether miR-340-5p directly regulates the proliferation of memory CD4^+^ T cells and what role it plays in the hepatic metabolic microenvironment.

Further association analysis suggested that Psat1 may be a potential regulatory target of miR-340-5p. Numerous studies have shown that the liver, as an important organ for maintaining systemic serine homeostasis, is closely associated with disease progression and metabolism. McBride et al. [55] used stable isotope tracing and liver-specific SHMT2 knockout mice to demonstrate that the liver can mediate the conversion of glycine to serine and subsequent metabolism through SHMT2, and that alterations in this process can significantly affect circulating glycine levels. In liver cancer, glycolysis drives serine metabolic reprogramming, conferring lenvatinib resistance [56]. In the MASH mouse model, liver serine/threonine kinase 38 (STK38) is upregulated and induces systemic inflammation and promotes insulin resistance [57]. Untargeted metabolomics studies have shown that the glycine/serine/threonine metabolic pathway is significantly enriched in hepatitis B virus-related hepatitis, suggesting that its dysregulation may be associated with viral replication and inflammation [58]. Another urine GC/MS-based metabolomics study also confirmed that glycine/serine/threonine metabolism is a significantly dysregulated pathway in patients with chronic hepatitis B [59]. It is worth noting that serine is also an important metabolite for maintaining T cell effector responses and adaptive immunity, promoting clonal expansion of T cells by facilitating one-carbon metabolism and the generation of biosynthetic intermediates [60,61]. Furthermore, extracellular serine also provides glycine and one-carbon units for proliferating T cells, serving as essential biosynthetic precursors for de novo purine nucleotide synthesis [62,63,64]. Maintaining sufficient flux of one-carbon units into the THF cycle is a hallmark of transformed cells, which rely on exogenous serine for de novo serine synthesis to sustain tumor growth. Although glycine can supply one-carbon units to the THF cycle through the glycine cleavage system, unlike other cell types, glycine cannot compensate for serine deficiency in T cells [65,66]. The expansion of sufficient effector T cells is key to the body’s protective immunity against invading pathogens [67]. Limiting serine availability during primary immunization impairs subsequent T cell responses, manifested as reduced pathogen clearance upon rechallenge and decreased reactive memory T cells. Ma et al. [60] significantly inhibited T cell expansion in vivo through dietary restriction of serine and glycine. Studies have shown that T cells significantly enhance glucose metabolism toward serine and glycine following antigen recognition, with specific upregulation of *Psat1*, whereas serine or glycine deficiency impairs T cell protein synthesis and cytotoxic effector function [68]. *Psat1*, as a key enzyme in the serine biosynthesis pathway, plays an important role in maintaining the metabolic homeostasis and immune function of T cells, especially CD4^+^ T cells [69,70]. A close association exists between *Psat1* expression and CD4^+^ memory T cell subsets, with elevated expression levels typically associated with increased infiltration or activation of memory CD4^+^ T cells [71]. Furthermore, proteomic studies showed that *Psat1* expression was significantly upregulated 72 h after activation of human primary CD4^+^ T cells [72]. Transcriptome WGCNA in this study revealed that *Psat1* is one of the most significantly downregulated and most stable genes in the NWS group. Meanwhile, molecular experiments showed that *Psat1* expression in liver tissue was significantly reduced, and immunofluorescence detection revealed a synchronous decrease in the level of memory CD4^+^ T cells in the liver. The above findings further support the potential association between altered Psat1 expression and abnormal memory CD4^+^ T cells. In vitro experiments confirmed that miR-340-5p mimics reduced *Psat1* expression in AML12 cells and primary CD4^+^ T cells, supporting the negative regulatory effect of miR-340-5p on *Psat1*. Based on the above results, we speculate that under the conditions of a high-fat, high-sugar diet and a high-temperature, high-humidity environment, the upregulation of miR-340-5p may interfere with serine metabolism-related immunometabolic reprogramming by targeting and inhibiting *Psat1* expression. The aforementioned regulatory process may further affect the maintenance and functional status of memory CD4^+^ T cells, and participate in the occurrence and development of chronic HBV infection-related hepatic inflammatory injury and immune imbalance.

It should be noted that this study focuses on the combined effects of dual exposure to a high-fat, high-sugar diet and a hot and humid environment on metabolic and immune homeostasis associated with chronic HBV infection, and thus still has certain limitations in the design of environmental exposure. Since the thermoneutral state of mice is not fixed at a single temperature but is influenced by factors such as circadian rhythm, strain, and husbandry conditions, most studies suggest that their thermoneutral point is approximately 30–32 °C [73]. Therefore, although the 25 °C environment used in this study falls within the conventional laboratory housing temperature range, it is still below the typical thermoneutral level for mice and may be accompanied by a certain degree of cold stress. In contrast, 32 °C is already close to the upper limit of the thermoneutral range for mice, and 95% relative humidity can further limit evaporative heat loss, making its effective heat load significantly higher than that of dry heat at 32 °C alone. Therefore, this model is more suitable as an experimental model for combined heat and humidity exposure, and should not be regarded as a direct or equivalent simulation of human heatwave exposure. Future studies could include a 32 °C and normal humidity group and incorporate control designs with different combinations of temperature and humidity to further distinguish the effects of temperature, humidity, and their interaction, thereby improving the accuracy of interpreting environmental exposure effects.

## 5. Conclusions

In summary, this study, through multi-omics integrative analysis and experimental validation, suggests that a high-fat and high-sugar diet combined with exposure to a high-temperature and high-humidity environment may be involved in the progression of CHB disease by affecting specific molecular pathways. We preliminarily identified miR-340-5p as a key upstream regulatory factor and speculated that it may target and inhibit Psat1 expression through a post-transcriptional regulatory mechanism, thereby affecting serine metabolism and the functional homeostasis of memory CD4^+^ T cells. Although the above findings provide clues for understanding the immunopathological mechanisms of HBV under the combined effects of dietary and environmental factors, the molecular regulatory mechanisms involved still require further functional experimental validation.

## Figures and Tables

**Table 1:** Macronutrient composition of the high-fat diet and the normal maintenance diet.

Nutrient	High-Fat Diet	Normal Maintenance Diet
Protein		
Amount (g/kg)	210	203.7
Energy (kcal/kg)	840	814.85
% of energy	0.2	0.215
Fat		
Amount (g/kg)	120	46.7
Energy (kcal/kg)	1050	420.69
% of energy	0.25	0.111
Carbohydrate		
Amount (g/kg)	570	638.6
Energy (kcal/kg)	2310	2554.46
% of energy	0.55	0.674
Energy density	4.2 kcal/g	3.79 kcal/g

**Table 2:** qPCR primer sequences of HBV-DNA and internal reference gene expression.

Primer Name	Sequence (5′ to 3′)
mus-HBV-F	TATCGCTGGATGTGTCTGCG
mus-HBV-R	GGTGCAATTTCCGTCCGAAG
mus-gapdh-DNA-F	CCCTTCCCACCCTGTTCATC
mus-gapdh-DNA-R	GCTCCTTGCCCTTCCAGATT

**Table 3:** Primer sequences.

Primer Name	Sequence (5′ to 3′)
U6-RT-R	AACGCTTCACGAATTTGCGT
U6-F	CTCGCTTCGGCAGCACA
Ctrl-minic	UUCUCCGAACGUGUCACGUTT
miR340-minic	UUAUAAAGCAAUGAGACUGAUU
mus-miR340-RT	GTCGTATCCAGTGCAGGGTCCGAGGTATTCGCACTGGATACGACAATCAG
mus-miR340-F	GCGCGTTATAAAGCAATGAGA
mus-miR340-R	AGTGCAGGGTCCGAGGTATT
m-GAPDH-F	TGACCTCAACTACATGGTCTACA
m-GAPDH-R	CTTCCCATTCTCGGCCTTG
m-Psat1-F	GAAGCCACCAAGCAAGTGGT
m-Psat1-R	GAGCACACTGATGCCGAGT

**Table 4:** Gene modules and gene counts.

Module	The Number of Genes
black	487
blue	1772
brown	1427
cyan	291
darkgreen	178
darkgrey	160
darkorange	133
darkred	194
darkturquoise	161
green	1052
greenyellow	365
grey	9
grey60	251
lightcyan	269
lightgreen	242
lightyellow	214
magenta	390
midnightblue	284
orange	150
pink	420
purple	387
red	552
royalblue	195
saddlebrown	36
salmon	328
skyblue	42
tan	339
turquoise	5096
white	42
yellow	1283

**Table 5:** Screening and Identification of Hub mRNAs Associated with memory CD4^+^ T cells.

Gene_Id	Gene_Name	log2(fc)	q Val	Abs (MM)
MSTRG.3523	NA	−1.877379 2846839	0.0000388874518077104	0.786924112719501
MSTRG.14095	NA	−1.06089 954465074	0.000108389460855815	1.7869241127195
ENSMUSG00000031327	Chic1	−1.043815 58329629	0.00330056167637715	0.732905683543491
MSTRG.9030	NA	−1.08152583025156	0.025167800627714	0.793397729789449
MSTRG.4059	NA	−1.18401967435188	0.000680979829345346	0.69537810666037
MSTRG.12601	NA	−1.18292163914748	0.0000638445433781192	0.953260665711152
MSTRG.13027	NA	−1.48480710668814	0.000884878800023153	0.675610439161703
MSTRG.9881	NA	−1.98144235519168	5.03797884866794 × 10^−14^	0.893962681635952
MSTRG.2393	NA	−1.03167060380523	0.0265598323874281	0.731746239535309
MSTRG.11849	NA	−1.04767556279642	0.00468488611862661	0.857175592844299
ENSMUSG00000037259	Dzank1	−1.17134387336418	0.00677670487423747	0.696156754587486
MSTRG.3219	NA	−1.14305589957865	0.0124642674422767	0.706656571422449
ENSMUSG00000033233	Trim45	−1.05910562192108	0.00604260480264202	0.829655929028307
ENSMUSG00000065987	Cd209b	−2.7494365991581	0.00348699844944567	0.696840973064723
MSTRG.11853	NA	−1.62116102687326	7.50038877557595 × 10^−8^	0.561266238588425
ENSMUSG00000026107	Nabp1	−1.07726889655668	0.016319030620509	0.655484286946041
MSTRG.12779	NA	−1.31621361540657	0.000325916922000284	0.860135789310515
ENSMUSG00000053950	Adnp2	−1.00041113242705	0.0000001432648724649	0.493266456203492
MSTRG.8659	NA	−1.3314737766243	0.000680979829345346	0.808355193965703
ENSMUSG00000050737	Ptges	−2.17654139877417	6.57827222398451 × 10^−8^	0.863974064368234
MSTRG.7126	NA	−1.40354630455814	0.0195168473849327	0.676033491556806
MSTRG.7642	NA	−2.3006643234258	3.39114728192541 × 10^−6^	0.856306132042947
MSTRG.10354	NA	−1.75963382789554	0.0000298537062638887	0.904316894535235
MSTRG.7853	NA	−1.36185684800824	0.0000147011458696774	0.626796074813878
MSTRG.7097	NA	−1.40274395855583	0.000430033546992353	0.728304968167133
ENSMUSG00000024640	Psat1	−1.41358905461572	0.00520684346622521	0.615090340208695
ENSMUSG00000029752	Asns	−1.9077625153421	2.83506862712286 × 10^−6^	0.482197134075772
ENSMUSG00000041658	Rragb	−1.34598020820735	0.0491870325305983	0.571702904061401
MSTRG.14256	NA	−1.03403629914286	0.000476075318983979	0.744602870335637
ENSMUSG00000029009	Mthfr	−1.35298842274803	0.00393990439244783	0.604630349220414
MSTRG.12897	NA	−1.76176042825166	6.55388260856891 × 10^−10^	0.892283207927299
ENSMUSG00000029086	Prom1	−1.09115784472872	0.0127219790694439	0.863541620222521
MSTRG.1961	NA	−1.24780697378669	0.0000212519844647005	0.719622270672438
MSTRG.1730	NA	−1.42947976556235	0.0124642674422767	0.836842317998379
MSTRG.209	NA	0.288701211227935	0.0844864598341708	0.767424420021254
ENSMUSG00000056880	Gadl1	−8.40490581678854	0.000882843129933808	0.680102965063326

## Data Availability

The M group dataset has been deposited in the China National Center for Bioinformation (CNCB) under project number PRJCA049371 and will be publicly available from 27 October 2027. The NWS group dataset has also been deposited in CNCB under project number PRJCA069010 and will be publicly available from 13 July 2028.

## Supplementary Materials

The following supporting information can be downloaded at: https://www.mdpi.com/article/10.3390/biology15201811/s1, Supplementary Table S1. Densitometric analysis of Western blot bands and relative fold change in protein expression. Supplementary Figure S1. Full-length, uncropped Western blot membranes with molecular weight markers. File S1: Original Western blot images for Figure 9.

## Abbreviations

The following abbreviations are used in this manuscript:

HBV	Hepatitis B Virus
CHB	Chronic Hepatitis B
cccDNA	covalently closed circular DNA
WGCNA	Weighted Gene Co-expression Network Analysis
