# Supplementary material for: Study on the Effects of Combined Exposure to a High-Fat, High-Sugar Diet and a High-Temperature, High-Humidity Environment on Adaptive Immunity in CHB Mice Based on Multi-Omics Analysis
Source: Biology (Basel). 2026 Oct 10;15(20):1811. doi: 10.3390/biology15201811 (PMC13701234; doi:10.3390/biology15201811)
Supplement: Supplementary file 1 [file biology-15-01811-s001.zip › Western blot.pdf]

Supplementary Table S1. Densitometric analysis of Western blot bands and relative fold change in protein expression

Sample	group	Target Protein (IntDen)	Loading Control (IntDen)	Ratio (Target/Loading Control)
NWS1	NWS	25633	339966	0.0754
NWS2	NWS	33891	286104	0.1185
NWS3	NWS	32116	219480	0.1463
M1	M	76288	208158	0.3665
M2	M	82069	274724	0.2987
M3	M	46384	275072	0.1686
